# Enhancement of Wear and Corrosion Resistance of Ti6Al4V Alloy through Hollow Cathode Discharge-Assisted Plasma Nitriding

**DOI:** 10.3390/ma17174386

**Published:** 2024-09-05

**Authors:** Hongyu Shen, Liang Wang

**Affiliations:** Department of Materials Science and Engineering, Dalian Maritime University, Dalian 116026, China; shen.hy.ol@outlook.com

**Keywords:** Ti6Al4V alloy, plasma nitriding, hollow cathode discharge, wear, corrosion

## Abstract

In order to improve the wear and corrosion resistance of Ti6Al4V alloy, a Ti-N compound layer was formed on the alloy by plasma nitriding at a relatively low temperature (750 °C) and within an economical processing duration (4 h), in a mixture of NH_3_ and N_2_ gases with varying ratios. The influence of the gas mixture on the microstructure, phase composition, and properties of the Ti-N layer was investigated. The results indicated that the thickness of the nitrided layer achieved in a mixed atmosphere with optimal proportions of NH_3_ and N_2_ (with a ratio of 1:2) was substantially greater than that obtained in an atmosphere of pure NH_3_. This suggests that appropriately increasing the proportion of N_2_ in the nitriding atmosphere is beneficial for the growth of the nitrided layer. The experiments demonstrated that the formation of the surface nitrided layer significantly enhances the corrosion and wear resistance of the titanium alloys.

## 1. Introduction

Owing to their superior mechanical properties, low density, and excellent corrosion resistance, titanium alloys find extensive applications across various industries, including the aerospace, medical, automotive, and industrial sectors. However, their low hardness and poor tribological characteristics constrain their broader utilization. Despite the inherently low hardness and wear resistance of titanium and its alloys, Ti-N (TiN and Ti_2_N) compounds exhibit high hardness, chemical stability, and wear resistance. Consequently, surface modification techniques are frequently utilized to deposit a Ti-N compound layer on these materials, thereby enhancing their hardness, wear resistance, and even corrosion resistance. The Ti-N compound layer serves as a protective barrier, reducing friction and wear by impeding direct contact between the alloy surface and the contacting material [1,2]. This results in enhanced durability and wear resistance, which is particularly advantageous in applications where the material is exposed to repetitive or high-load contacts. The primary techniques employed for this purpose include deposition and diffusion processes such as plasma nitriding and the physical vapor deposition (PVD) of hard coatings [3,4,5,6,7,8]. The benefits of PVD include relatively low deposition temperatures, a high deposition efficiency, and the flexibility to adjust the coating composition, which allows for a wide range of coating properties. Nolan et al. compared the wear resistance of titanium nitride thin films deposited on Ti-6Al-4V alloy using PVD and plasma nitriding processes. They reported that the TiN film exhibited a lower resistance to applied normal loads in sliding wear due to the reduced mechanical support from the underlying substrate, despite its higher hardness. Their research clearly illustrated the beneficial effects of substrate hardening resulting from the nitrogen diffusion layer [9]. Ceschini et al. deposited a 3.5 µm thick TiN coating on a Ti6Al4V alloy using PVD. The friction and wear of the alloy were significantly reduced by the presence of the thin PVD coatings; however, the durability of these coatings was strongly dependent on the applied loads [10]. Compared to PVD, nitriding offers the distinct advantage of lacking a precise interface between the compound layer and the substrate. This implies that under external loads, there is no delamination of the layer due to insufficient bonding strength between the layer and the substrate. Additionally, nitriding does not exhibit a pronounced line-of-sight effect, which allows for the processing of complex-shaped workpieces. Wilson et al. performed the surface modification of Ti6Al4V using plasma nitriding, the physical vapor deposition (PVD) of TiN coatings, and nitrogen ion implantation, and they compared the effects of these treatments on tribological properties. Both plasma nitriding and TiN coatings substantially enhanced the surface hardness and wear resistance of the alloy, although the nitrided layer exhibited a higher load-carrying capacity than the coating [11]. Sobiecki et al. investigated the effects of oxynitriding on titanium alloys by treating them at a constant temperature of 900 °C for varying durations of 3, 6, and 12 h. A composite layer consisting of titanium oxides and titanium nitrides was formed on the surface of the alloy. The resulting layer significantly enhanced the wear resistance of the titanium alloys and also improved their corrosion resistance. Furthermore, it was found that an increase in treatment duration was directly proportional to an increase in wear resistance. Specifically, the wear volume of the samples treated for 12 h was observed to be four times lower than that of the samples treated for only 3 h [12]. Yildiz et al. performed plasma nitriding on titanium alloys, which were treated for 1–4 h within a temperature range of 650–750 °C. Compared to the un-nitrided samples, the wear and corrosion resistance of the titanium alloys were significantly improved after nitriding [13].

Due to the very low diffusion coefficient of nitrogen in titanium alloys, particularly in Ti-N compounds [14,15,16], the compound layers formed by plasma nitriding on titanium alloys are generally quite thin, especially at lower temperatures. According to diffusion theory, the primary factors influencing the thickness of the nitrided layer include temperature, time, gas composition, and other parameters. The appropriate selection of nitriding conditions, such as temperature and the nitrogen–hydrogen gas ratio, permits the optimization of the nitrided layer’s properties, thereby enhancing the material’s performance in tribological applications [17]. Hosseini et al. reported on research conducted on the plasma nitriding of a Ti6Al4V alloy at temperatures ranging from 700 to 850 °C for durations of 2 to 10 h. They found that both increasing the plasma nitriding temperature and extending the process time significantly improved the structural and tribological characteristics of the alloy. After nitriding at various temperatures for 10 h, the resultant compound layer was found to be approximately 1 to 2 µm thick [18]. Chen et al. nitrided Ti6Al4V using a nitrogen–hydrogen mixed gas at temperatures ranging from 600 to 900 °C for 20 h. The ratio of nitrogen to hydrogen in the mixed gas and the temperature significantly influenced the thickness of the compound layer. A compound layer with a thickness of approximately 5 µm was achieved after nitriding at 700 °C for 20 h [6].

In order to obtain a Ti-N compound layer thicker than 2 µm on a Ti6Al4V alloy at a relatively low temperature within a reasonable processing time, the current study focuses on examining the influence of the ammonia-to-nitrogen ratio in the mixture on the plasma nitriding of the Ti6Al4V alloy at a temperature of 750 °C for 4 h. The nitrided specimens were analyzed using scanning electron microscopy (SEM), X-ray diffraction (XRD), microhardness testing, and electrochemical measurements, as well as wear and friction tests.

## 2. Experimental Procedure

**Figure 1:**
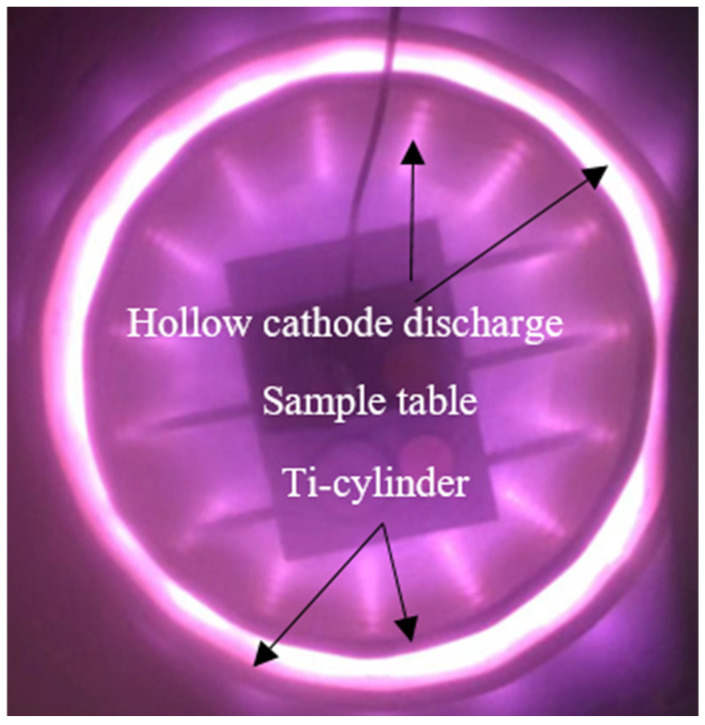
Photograph of hollow cathode discharge for nitriding processing.

The titanium alloy utilized in this study is Ti-6Al-4V, possessing a chemical composition with the proportions (in wt.%) Al (5.5–6.5), V (3.5–4.3), and the balance being Ti. Specimens were excised from 2 mm thick cold-rolled sheets of the titanium alloy and cut to dimensions of 10 mm × 10 mm. Prior to nitriding, the sample surfaces were polished sequentially with silicon carbide papers and then subjected to metallographic polishing. The nitriding process was conducted at a temperature of 750 °C for a period of 4 h, under an atmosphere consisting of pure ammonia, as well as mixtures of ammonia and nitrogen at ratios of 1:1 and 1:2, using a hollow cathode discharge-assisted plasma nitriding system [19]. The hollow cathode discharge system consists of two titanium metal cylinders with different diameters. To ensure a stable hollow cathode discharge at the selected operating pressure, holes with a diameter of approximately 8–10 mm are uniformly distributed on the surface of the cylinders, and the gap between the two cylinders is also maintained at 8–10 mm. In this study, a photograph of hollow cathode discharge and the setup for plasma nitriding is shown in Figure 1.

The surface morphology and compositional analysis of the samples were meticulously examined utilizing a scanning electron microscope (SEM; SUPRA 55, Zeiss, Germany), which was equipped with an energy-dispersive spectrometer (EDS; Ultim Max, Oxford, UK). X-ray diffraction (XRD) experiments were performed on a PANalytical Empyrean diffractometer (Malvern Panalytical, Almelo, Netherlands) using a conventional Bragg–Brentano configuration and glancing incidence X-ray diffraction (GIXRD) with a fixed incidence angle of 0.5, 3, and 5° using Cu K_α_ radiation (λ = 1.54 Å). The tube acceleration voltage and current used were 40 kV and 30 mA, respectively.

The surface hardness of both untreated and plasma-nitrided Ti6Al4V alloys was assessed using a MH-6 Vickers microhardness tester (Hongxin Electronic Technology Co., Ltd., Shanghai China) at an applied load of 0.5, 1.0, and 2.0 N.

For a given load, the penetration depth depends on the surface hardness of the materials measured. For the samples measured in this study, the penetration depth was in a range of 1.46 to 6.31 μm. 

To ensure accuracy, each hardness measurement was replicated five times, and the mean value was subsequently determined for more reliable results.

The corrosion resistance of the samples was evaluated through potentiodynamic polarization tests conducted in a 3.5 wt% NaCl solution. These tests utilized a three-electrode electrochemical cell coupled with a CHI660C electrochemical workstation. Platinum foil acted as the counter electrode, a saturated calomel electrode (SCE) served as the reference electrode, and the sample itself functioned as the working electrode. Potentiodynamic polarization tests were performed within a potential range of −0.8 V to 2.0 V, with a scanning rate of 1 mV/s.

In order to evaluate the tribological properties, experiments were performed on a HT-600 ball-on-disc tribometer (Zhong Ke Kaihua, Lanzhou, China) (in air with a relative humidity of approximately 38% at ambient temperatures, without any lubricant. The specimen rotated at a speed of 0.21 m/s against a pin made of an AISI 51200 bearing steel ball (Tianjin bearing factory, Tianjin China, HRC 63-65) of 4 mm diameter under a normal load of 3 and 8 N. The sliding time was fixed at 60 min, resulting in a sliding distance of approximately 754 m. During the test, the friction coefficient was continuously monitored as a function of sliding time. To investigate the wear behavior, the width, topography, and composition of the sliding tracks were analyzed using scanning electron microscopy (SEM) and confocal laser scanning microscopy (CLSM-Olympus OLS4000, Tokyo, Japan). These advanced imaging techniques provided detailed insights into the wear mechanisms and the resulting surface alterations. Recently, a new device named GelSight Max (GelSight, Waltham, USA) has been used for the topographic measurement of stone tool surfaces [20].

## 3. Results and Discussion

**Figure 2:**
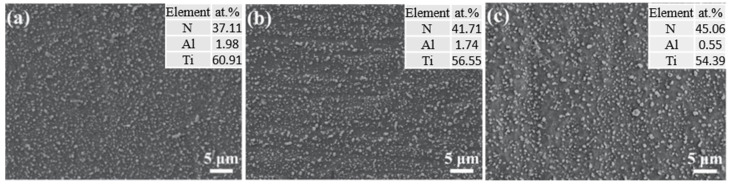
SEM surface micrographs of Ti6Al4V samples nitrided at 750 °C for 4 h in (**a**) NH_3_, (**b**) NH_3_-N_2_ (1:1), and (**c**) NH_3_-N_2_ (1:2).

Figure 2 depicts the scanning electron microscopy (SEM) surface morphology and the composition as measured by the energy-dispersive X-ray spectroscopy (EDX) of the titanium alloy following nitriding at 750 °C for 4 h under varying atmospheres.

As evident from the images, particles of diverse sizes, extending from the nanometer to the micrometer scale, are homogeneously dispersed across the surface. This type of morphology is frequently encountered on the surfaces of physical vapor deposition (PVD) coatings [21,22,23] and nitrided layers produced by plasma or gas nitriding processes. For plasma nitriding, the sample being treated is often connected to the discharge cathode. Under the action of the electric field, the positive ions in the plasma produce an intense ion bombardment and sputtering on the cathode. The particulate matter on the sample surface is primarily formed by the re-deposition of material sputtered from the surface. The granular material on the surface of the sample after gas nitriding is due to the formation of nitrides [24,25]. EDX spectral analysis reveals that the nitrogen content on the surface indicates the formation of a Ti-N compound layer on the titanium alloy’s surface. Additionally, the surface nitrogen concentration increases in proportion to the increment in nitrogen content within the nitriding atmosphere. The values of these nitrogen concentration are all within the scope of the composition for the TiN compound (above 28 at.%).

**Figure 3:**
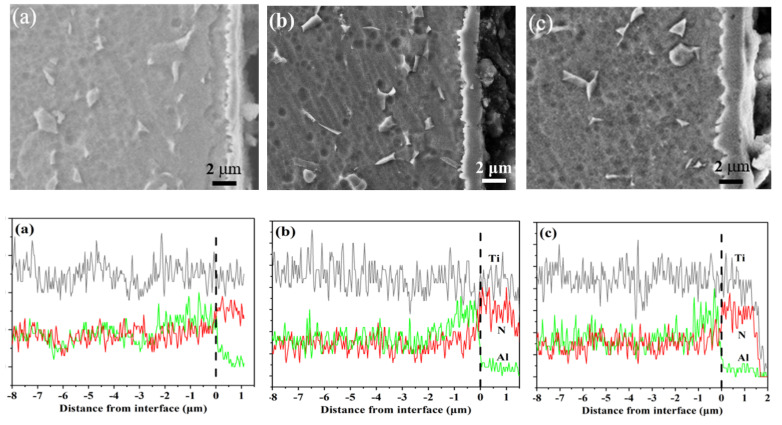
Cross sectional SEM images of the nitrided layers and an EDS elemental line analysis along the cross-sections of the samples: (**a**) nitrided in NH_3_, (**b**) nitrided in NH_3_-N_2_ (1:1), and (**c**) nitrided in NH_3_-N_2_ (1:2).

SEM images of the cross-sections of the nitrided samples are depicted in Figure 3, elucidating the influence of three atmospheres on the nitriding process.

Despite an equivalent temperature and nitriding duration, a pronounced disparity in thickness among the three Ti-N layers is apparent, with the layer formed in ammonia exhibiting a thickness of approximately 1.1–1.4 μm. Owing to the limited diffusion coefficient of nitrogen in both the titanium alloy matrix and Ti-N compounds, the Ti-N layer remains relatively thin even after extended nitriding at lower temperatures. The plasma nitriding of TC4 alloy at 500 °C for 20 h within a pure ammonia atmosphere at various pressures yielded a compound layer with a thickness ranging from 0.5–1.3 μm [26]. Gisele et al. achieved a 1 μm thick compound layer on a titanium alloy through plasma nitriding at 690 °C for 4 h in a mixed atmosphere of Ar (45.5%) + N_2_ (45.5%) + H_2_ (9%) [27]. Nitriding Ti-6Al-4V at 750 °C and 850 °C for 24 h in an atmosphere with a N_2_:H_2_ ratio of 6:4 resulted in the formation of compound layers with thicknesses of approximately 2 μm and 5 μm, respectively [28]. In a dilute nitriding atmosphere containing 3% N_2_, a 1.9 μm thick compound layer was produced on a titanium alloy following plasma nitriding at 600 °C for 24 h [29]. Szymkiewicz et al. reported a 0.7 μm thick compound layer formed on Ti6Al4V after nitriding at 740 °C for 4 h using active screen plasma nitriding in a N_2_ and H_2_ mixture [30]. A Ti-N compound layer with a thickness of 1.5 μm was formed by plasma nitriding at 730 °C for 20 h in pure nitrogen and at 760 °C for 6 h in a gaseous mixture comprising 80% nitrogen and 20% hydrogen [31,32]. Increasing the nitrogen content to achieve an NH_3_-to-N_2_ ratio of 1:1 led to a modest increase in the thickness of the compound layer, which reached approximately 1.5–1.9 μm. When the NH_3_:N_2_ ratio was increased to 1:2, the Ti-N compound layer further increased to about 1.7–2.2 μm. As observed from the energy-dispersive X-ray (EDX) line scanning, which reflects the trend of element distribution, there is a concentration of Al at the interface between the compound layer and the matrix. This suggests that during the nitriding process, the formation of the compound layer induced the diffusion of aluminum into the matrix, corroborating the findings of other studies [33,34]. Hosseini et al. postulated that the formation of the Ti-N compound layer increases the chemical potential of Al within the system, facilitating its diffusion. However, the Ti-N compound layer impedes this diffusion, resulting in the accumulation of aluminum elements at the interface between the compound layer and the matrix [18].

**Figure 4:**
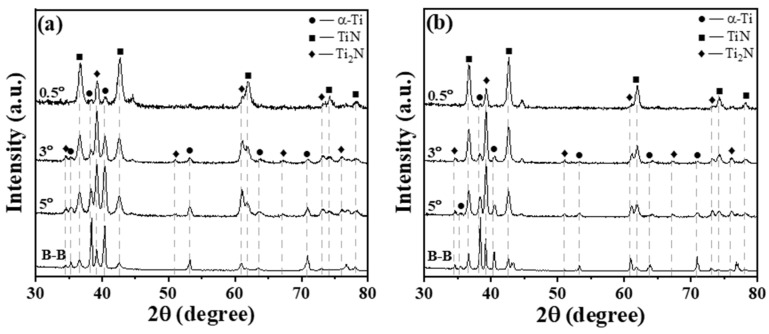
XRD patterns for Ti6Al4V with B-B configuration and GIXRD patterns of nitrided Ti6Al4V at fixed incident angles of 5°, 3° and 0.5°: (**a**) nitrided in NH_3_, (**b**) nitrided in NH_3_-N_2_ (1:2).

Figure 4 presents conventional XRD and GXRD patterns of samples nitrided in NH_3_ and NH_3_-N_2_ (1:2).

From the patterns obtained by the B-B model, the samples nitrided under the two distinct atmospheres all exhibit diffraction peaks attributable to the nitride phases Ti_2_N and TiN [9,14,35,36]. Typically, in accordance with the Ti-N phase diagram, a nitrogen-containing solid solution forms initially, followed by the formation of Ti_2_N and TiN as the nitrogen concentration surpasses its maximum solid solubility for nitrogen diffusion processing [17]. Thus, the super surface is composed of TiN, and the layer following it should be Ti_2_N. For the sample nitrided in NH_3_, the peaks corresponding to α-Ti from the substrate are much stronger than those from the compound layer, which is only about 1 μm thick. As the thickness of the compound layer increases, the intensity of the diffraction peaks corresponding to TiN also intensifies. With the compound layer increased to about 2 μm for the sample nitrided in NH_3_-N_2_ (1:2), the intensity of peaks from the substrate decreases. GIXRD patterns of the nitrided sample provided the phase information coming from the different depth below the surface of the nitrided layer. The intensity for various phases changed with the incidence angle of the X-rays. With a fixed incidence angle of 0.5°, the peaks from TiN were much higher than those of Ti_2_N. As the incidence angle increased, the intensity of the Ti_2_N peaks gradually became stronger because the penetration of the X-rays increased. These observations demonstrate the forming sequence of the Ti-N compound layer for nitrogen diffusion processing. Combined with the surface composition measured by EDX shown in Figure 2 and the conventional XRD result, it is clear that the compound layer is composed of TiN and Ti_2_N.

**Figure 5:**
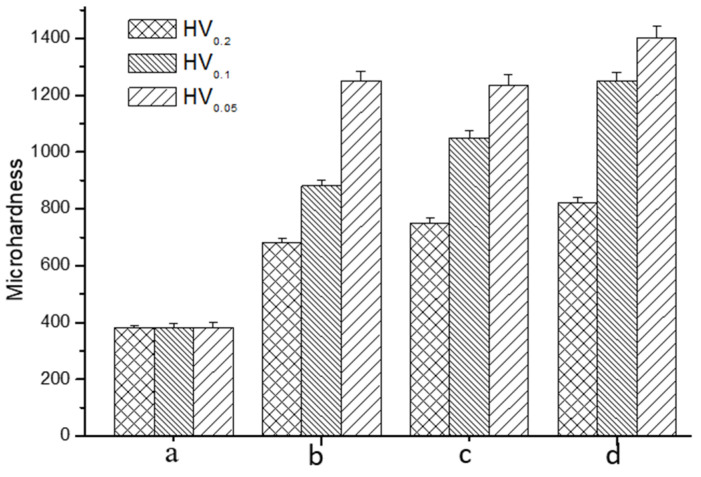
Surface microhardness of the samples measured with three loads: (a) untreated, (b) nitrided in NH_3_, (c) nitrided in NH_3_-N_2_ (1:1), and (d) nitrided in NH_3_-N_2_ (1:2).

Microhardness testing with three different loads demonstrated a considerable enhancement in the surface hardness of the titanium alloy subsequent to the formation of a nitrided layer (Figure 5).

It is evident that the applied load during testing significantly influenced the measured surface hardness, especially for the nitrided in NH_3_. This is primarily attributed to the fact that the nitriding process resulted in the formation of a relatively thin compound layer, thereby necessitating consideration of the effects of the underlying matrix on the hardness measurement. The sample treated with NH_3_ exhibited a hardness of 880 HV_0.1_, which is more than twice the 380 HV_0.1_ of the untreated alloy. In comparison, the sample nitrided using a mixture of NH_3_ and N_2_ at a ratio of 1:2 demonstrated a surface hardness above 1200 HV_0.1_.

**Figure 6:**
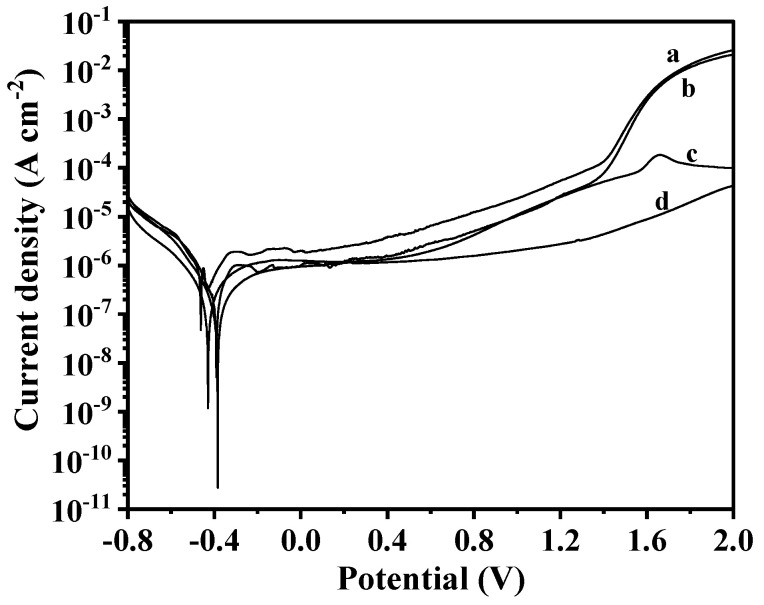
Polarization curves measured in 3.5% NaCl solution for samples that were (a) untreated, (b) nitrided in NH_3_, (c) nitrided in NH_3_-N_2_ (1:1), and (d) nitrided in NH_3_-N_2_ (1:2).

Figure 6 depicts the electrochemical anodic polarization curves obtained in a 3.5% NaCl solution for both the un-nitrided and nitrided samples.

All the samples exhibited passive behavior, and the maintenance current density decreased with the increasing thickness of the compound layer. Compared to the un-nitrided sample, the corrosion potential (E_corr_) of the nitrided samples exhibited a slight increase, whereas the corrosion current density (I_corr_) significantly decreased. As the polarization potential increased, the I_corr_ for both the un-nitrided sample and the sample nitrided in NH_3_ showed a gradual increase, with the trend of change being essentially consistent between the two. Upon reaching a potential of 1.5V, the I_corr_ displayed a rapid increase, indicating the onset of pitting corrosion. However, the sample nitrided in an NH_3_-N_2_ (1:2) atmosphere demonstrated an exceptionally slow increase in I_corr_ with an applied voltage, remaining in a stable passive state within the potential range of 0 to 1.2 V, with the I_corr_ at 1 μA. Even when the voltage was elevated to 2 V, the I_corr_ only reached approximately 50 μA, which was more than three orders of magnitude lower than that of the un-nitrided sample. These results suggest that the corrosion resistance of the titanium alloy can be substantially enhanced by the formation of a Ti-N compound layer.

**Figure 7:**
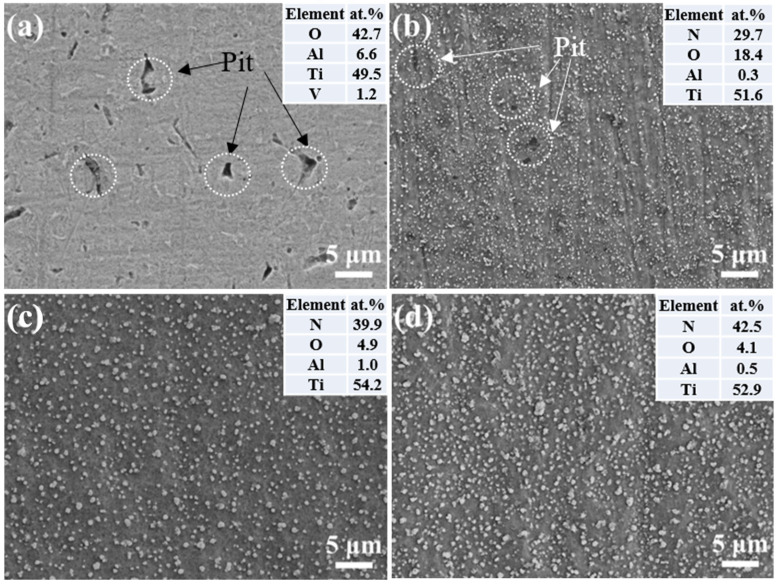
Surface morphology of sample after being corroded for (**a**) untreated, (**b**) nitrided in NH_3_, (**c**) nitrided in NH_3_-N_2_ (1:1) and (**d**) nitrided in NH_3_-N_2_ (1:2) samples.

The surface morphology of the samples following the corrosion test is illustrated in Figure 7. The surface of the un-nitrided sample exhibits numerous corrosion pits of varying shapes and sizes, confirming the occurrence of pitting corrosion on the sample surface. The sample nitrided in NH_3_ also reveals visible corrosion pits, although their number and size are notably diminished. In contrast, the samples nitrided in a mixed atmosphere exhibit no apparent changes on their surfaces after corrosion, indicating a significant resistance to corrosive attack. The absence of discernible corrosion identifies that the Ti-N layer formed on the surface is stable, leading to an inert state for Ti6Al4V following plasma nitriding. The formation of thicker Ti-N compound layers is helpful in improving the corrosion and wear resistance properties of titanium alloys [13].

This indicates that the formation of the nitrided layer significantly improves the corrosion resistance of titanium alloys, but the thickness of the nitrided layer is too thin to provide better protection for the substrate. For samples nitrided in a mixed gas atmosphere, the thickness of the formed nitrided layer is significantly increased, and the ability to resist corrosion by corrosive media is also correspondingly enhanced.

**Figure 8:**
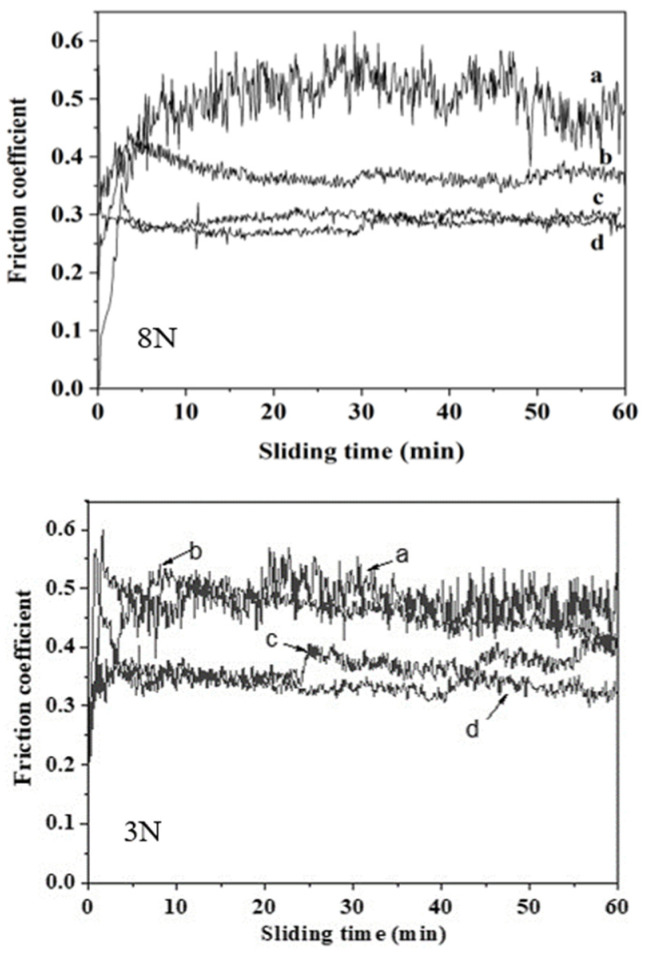
Friction coefficient of untreated and nitrided samples under 3N and 5N load: (a) untreated, (b) nitrided in NH_3_, (c) nitrided in NH_3_-N_2_ (1:1), and (d) nitrided in NH_3_-N_2_ (1:2).

The variation in the friction coefficient over sliding time for both the untreated and nitrided titanium alloy under an applied load of 3 and 8 N is depicted in Figure 8.

For the untreated sample, the friction coefficient fluctuated within a range of 0.45 to 0.55, following an initial continuous increase from the beginning of the test. The significant fluctuation amplitude suggests that the titanium alloy underwent processes of adhesion–separation and plowing during sliding against the friction pair, resulting in severe wear. A high coefficient of friction indicates greater resistance to sliding between the friction pairs, primarily due to the resistance caused by adhesion and plowing. This process hinders the friction and wear from achieving a stable state. Compared to the untreated titanium alloy, the friction curve of the sample nitrided in NH_3_, besides having smaller fluctuations, showed little difference in the magnitude of the friction coefficient under a load of 3N. All the samples that underwent nitriding showed a reduction in friction coefficient under an 8N load. In particular, the samples nitrided in atmospheres with ammonia to nitrogen ratios of 1:1 and 1:2 exhibited a significant decrease in the friction coefficient to around 0.3. This indicates that different wear mechanisms were operative on the samples before and after plasma nitriding. B. Januszewicz reported a similar result, with the friction coefficient decreasing from 0.7 to 0.3 for a Ti6Al4V alloy nitrided at 850 °C [33].

**Figure 9:**
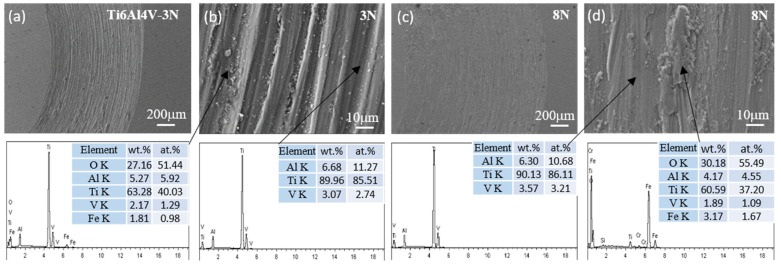
SEM micrographs and chemical composition of the wear scar on untreated Ti6Al4V under a load of 3N (**a**,**b**) and 8N (**c**,**d**).

**Figure 10:**
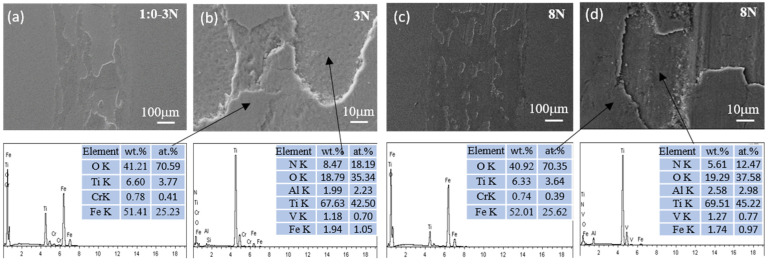
SEM micrographs and chemical composition of the wear scar on Ti6Al4V nitrided in NH_3_ under a load of 3N (**a**,**b**) and 8N (**c**,**d**).

**Figure 11:**
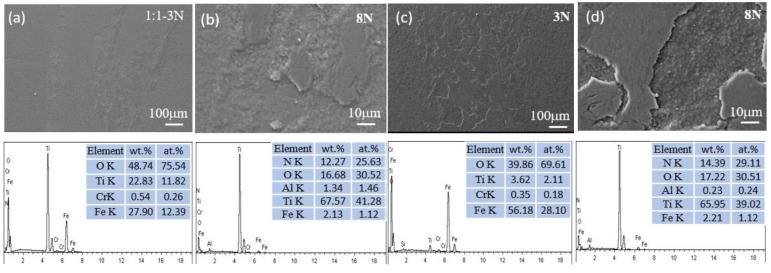
SEM micrographs and chemical composition of the wear scar on Ti6Al4V nitrided in NH_3_-N_2_ (1:1) under a load of 3N for (**a**,**b**) and 8N for (**c**,**d**).

**Figure 12:**
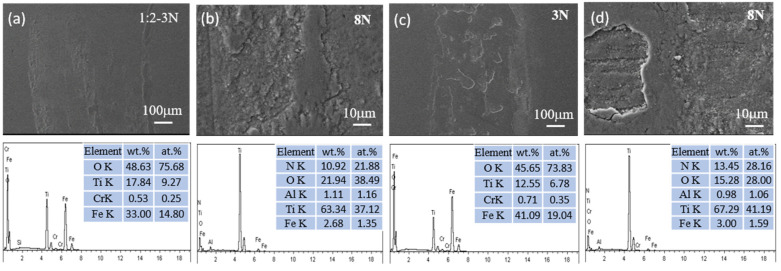
SEM micrographs and chemical composition of the wear scar on Ti6Al4V nitrided in NH_3_-N_2_ (1:2) under a load of 3N for (**a**,**b**) and 8N for (**c**,**d**).

The SEM micrographs and EDS measurements depicted in Figure 9, Figure 10, Figure 11 and Figure 12 present the wear tracks for both untreated and nitrided samples following a sliding duration of 60 min.

Compared to the 3N load, the wear scar width significantly increased under the 8N load, ranging from about 1000 to 1400 μm. At low magnification, the wear track on the untreated titanium alloy reveals numerous grooves after sliding under both loads. At high magnification, a considerable amount of wear debris and plastic deformation becomes evident, revealing more pronounced surface damage and material loss. These debris consist of oxides of Ti containing Al, Fe, and V, indicative of severe wear processes involving plowing and tearing.

Compared to the un-nitrided sample, a thin layer that was entirely distinct from the nitrided layer was present on the sample surface, partially overlaying the wear tracks. EDS analysis confirmed that this layer was primarily composed of iron oxides. The wear surfaces of the three nitrided samples exhibited a relative likeness in appearance. The wear scar widths were approximately 300 μm under the 3N load and 450 μm under the 8N load for the nitrided samples. No discernible grooves and plastic deformation were observed on the worn surfaces of the nitrided samples. This is attributed to the altered physical and chemical properties and the increased surface hardness associated with the formation of the Ti-N compound layer. The surface hardness was elevated from 380 HV for the bare titanium alloy to 880–1200 HV for the nitrided samples, which is sufficiently hard to prevent plowing by the counterface, despite the hardness of bearing steel being approximately 800 HV.

During the sliding contact, trace amounts of iron from the bearing steel were transferred to the surface of the Ti-N layer. Then, a thin oxide layer was formed under the influence of frictional force and heat. Once established, this layer effectively acted as a barrier between the bearing steel and the nitrided titanium alloy surface, mitigating direct contact and reducing the friction coefficient due to the layer’s own low friction properties. As a consequence, the worn surfaces of all the nitrided samples displayed characteristic signs of mild wear, such as the presence of an oxidized layer and a smoother appearance. Consequently, the transfer of adhesive material from the steel to the nitrided sample was a predominant mechanism in the sliding contact. The tribological behavior and wear dynamics were likely governed by the oxidation of the transferred iron. The enhancement in wear resistance can be ascribed to the presence of a compound layer with sufficient thickness, coupled with the higher hardness of this layer. Anandan et al. reported that samples nitrided at 800 °C for 2 h exhibited a wear reduction of one order of magnitude compared to un-nitrided samples under identical wear conditions. Furthermore, the friction coefficient of the nitrided samples was also reduced, indicating improvements in both wear resistance and friction properties. The decrease in wear and friction can be attributed to the formation of a thicker and harder nitrided layer on the surface of the Ti6Al4V alloy [7]. Wilson et al. observed material transfer from AISI 52100 balls to the surface of a nitrided sample, and the calculated wear rate was negative, indicating a net transfer of material from the softer ball to the harder, nitrided sample [11].

**Figure 13:**
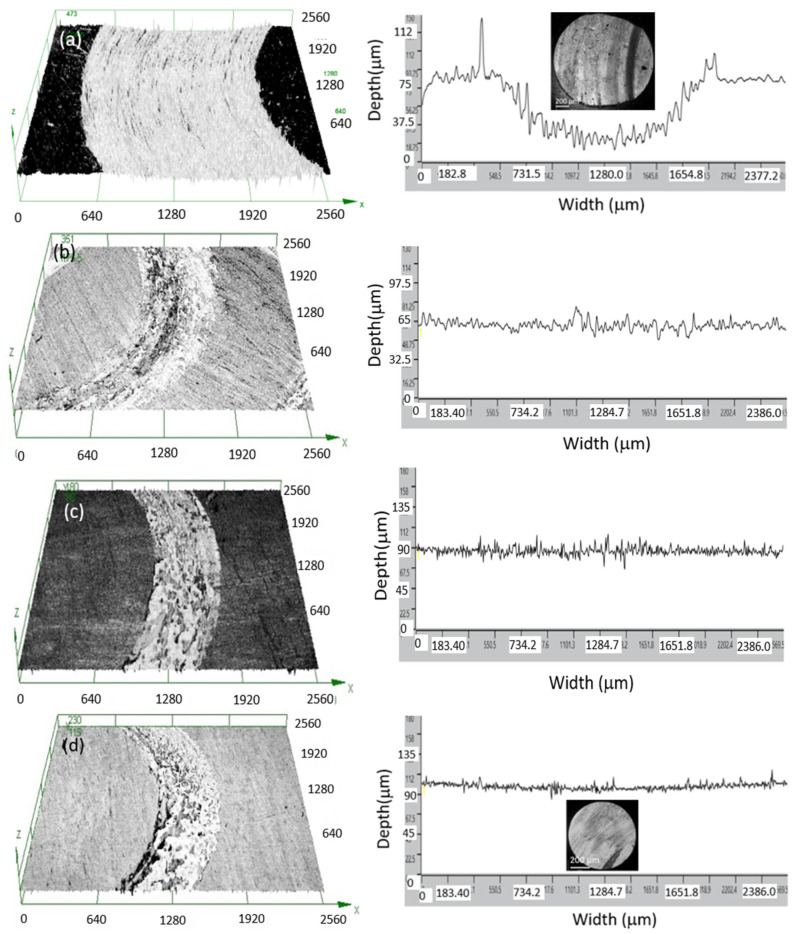
Three-dimensional profilometry images and 2D line scans of the wear tracks after sliding against bearing steel balls at a load of 8 N for 60 min formed on Ti6Al4V alloy that was (**a**) untreated, (**b**) nitrided in NH_3_, (**c**) nitrided in NH_3_-N_2_ (1:1), and (**d**) nitrided in NH_3_-N_2_ (1:2).

Three-dimensional profilometry images and 2D line scans of the wear tracks after sliding against bearing steel balls at a load of 8 N for 60 min formed on untreated and nitrided Ti6Al4V alloy are shown in Figure 13.

Deep wear grooves are evident throughout the wear track of the un-nitrided sample. The depth and width of the wear scar on the untreated sample were approximately 65 μm and 1400 μm. The width of the wear scar was clearly reduced for the nitrided samples. It was about 650 μm for the sample nitrided in NH_3_, 565 μm for that nitrided in NH_3_-N_2_ (1:1), and 525 for that nitrided in NH_3_-N_2_ (1:2). Compared to the untreated samples, the width of the wear tracks was reduced by more than a factor of two. Due to the presence of the oxide film layer, no measurable depth to the wear tracks was detected on all the nitrided samples. The plasma nitriding treatment significantly improved the substrate’s wear resistance, resulting in material transfer from the steel ball to the surface of the nitrided titanium alloy. A transition in wear behavior from severe plowing to a polishing–rubbing type is likely to be observed. The images of scars left on the counterparts after sliding against untreated and nitrided samples are inserted in Figure 13. The surface of the wear scar left on the steel ball after sliding against the untreated sample is relatively rough, with obvious scratches and adhesion phenomena. In contrast, the wear scar surface left after sliding against the sample nitrided in NH_3_-N_2_ (1:2) is much smoother and brighter, indicating a decrease in the friction coefficient.

## 4. Conclusions

(1) The growth of the Ti-N compound layer was enhanced by the use of an NH_3_-N_2_ mixture during the plasma nitriding of the Ti6Al4V alloy. After nitriding at 750 °C for 4 h in an NH_3_-N_2_ (1:2) atmosphere, a Ti-N compound layer approximately 2 μm thick was formed on the Ti6Al4V alloy.
(2) The Ti-N compound layers are typically much harder than the titanium alloy substrate. In comparison to the un-nitrided titanium alloy, samples nitrided in an NH_3_-N_2_ mixture exhibited the complete elimination of stick–tear and plowing wear throughout the duration of the test, resulting in undetectable wear loss when in contact with bearing steel.
(3) The oxide layer formed on the wear track by the transfer of material from the counterpart steel ball reduced the average friction coefficient from approximately 0.55 to 0.30 under an 8N load.
(4) The formation of the Ti-N layer not only enhances the hardness and wear resistance of the titanium alloy but also increases its corrosion resistance.

## Data Availability

The original contributions presented in the study are included in the article, further inquiries can be directed to the corresponding author.
